# Are kuravirus capsid diameters quantized? The first all-atom genome tracing method for double-stranded DNA viruses

**DOI:** 10.1093/nar/gkad1153

**Published:** 2023-12-12

**Authors:** Samuel Coulbourn Flores, Michal Malý, Dominik Hrebík, Pavel Plevka, Jiří Černý

**Affiliations:** Swedish University of Agricultural Sciences, Ulls Väg 26, Uppsala, and Stockholm University, Tomtebodavägen 23A, Solna, Sweden; Institute of Biotechnology of the Czech Academy of Sciences, Prumyslova 595, Vestec 25250, Czech Republic; Central European Institute of Technology, Kamenice 753/5, Brno, Czech Republic; Central European Institute of Technology, Kamenice 753/5, Brno, Czech Republic; Institute of Biotechnology of the Czech Academy of Sciences, Prumyslova 595, Vestec 25250, Czech Republic

## Abstract

The revolution in cryo-electron microscopy has resulted in unprecedented power to resolve large macromolecular complexes including viruses. Many methods exist to explain density corresponding to proteins and thus entire protein capsids have been solved at the all-atom level. However methods for nucleic acids lag behind, and no all-atom viral double-stranded DNA genomes have been published at all. We here present a method which exploits the spiral winding patterns of DNA in icosahedral capsids. The method quickly generates shells of DNA wound in user-specified, idealized spherical or cylindrical spirals. For transition regions, the method allows guided semiflexible fitting. For the *kuravirus* SU10, our method explains most of the density in a semiautomated fashion. The results suggest rules for DNA turns in the end caps under which two discrete parameters determine the capsid inner diameter. We suggest that other kuraviruses viruses may follow the same winding scheme, producing a discrete rather than continuous spectrum of capsid inner diameters. Our software may be used to explain the published density maps of other double-stranded DNA viruses and uncover their genome packaging principles.

## Graphical Abstract

**Graphical Abstract:**
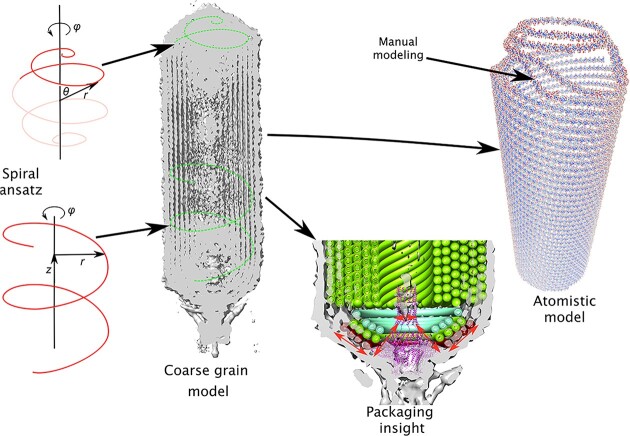

## Introduction

### Models of spiral DNA genome packaging for icosahedral viruses

Genome packaging in double-stranded (ds) DNA viruses is an attractive topic because its regularity and similarity across many viruses, including pathogens such as HSV-1 (1,2) suggests tractability and wide applicability (3). Early simplified models of DNA packaging into icosahedral virus capsids present DNA as comprising closed, flat, concentric, circular rings, all perpendicular to the virus capsid axis (4). These were appealing as they could be modeled with simple physical equations and easily predict quantities like pressure based on DNA persistence length.

One step up from this are coaxial spool models (3). A spiral proposed by (5) would follow a spherical shell (6). Later workers wrote software to test this idea on CryoEM densities (3), but did not build 3D models. Coarse-grained models along these lines have been built (7) for simulations. Outside the present work, we know of no publicly accessible (3) software to trace spiral genomes (spherical (6) or cylindrical, automated or otherwise) in density maps, and none at all that can build all-atom models of such spirals. Other packaging models (not treated in this work) include liquid-crystal (8) and toroidal (9).

**Figure 1.**
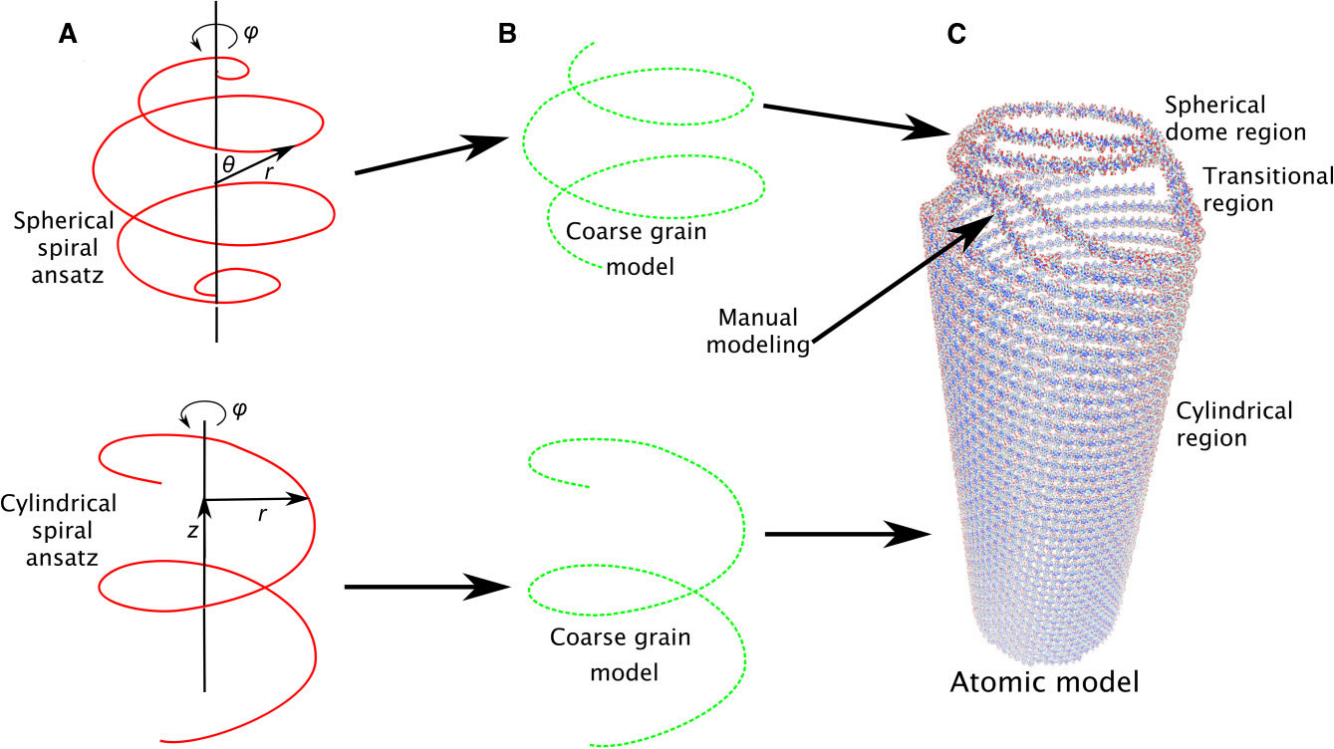
Genome tracing procedure for cylindrical viruses with spherical-dome end caps, like SU10. (**A**) MMB supports spherical and cylindrical spirals. (images from Wikipedia, under Creative Commons license). (**B**) In a coarse grained run, a single atom represents each base pair. Fitting energy is computed, against a user-provided density map. The spiral parameters can be optimized using a loop. Multiple coarse-grained spirals can be generated simultaneously. (**C**) Once parameters are optimal, the coarse-grained can be converted to an all-atoms model.

We present a tool to trace genomes following a spherical or cylindrical spiral under the coaxial (or, with adaptation, concentric (9)) spool model. In a first, coarse-grained step, a single pseudoatom represents each base pair, and many such pseudoatoms form a spiral. The spiral parameters are scanned to optimize the pseudoatoms’ summed fitting energy against the experimental density map (Figure 1). The coarse grained step is fast, and produced most of the conclusions in this work.

To translate the coarse to a fine grained model, we start from the idealized structure of two nucleotides in a Watson Crick/Watson Crick/Cis base pair (10). To this pair of nucleotides we apply simple translations and rotations which progressively generate dsDNA in a spiral conformation, where the center of the pair follows the (coarse grained) pseudoatom locations.

As the above paired nucleotides were translated-rotated rigidly, the bond connecting successive residues along both helical strands is to some degree compressed on the inside of the spiral, and extended on the outside. To correct this we perform a restrained minimization using OpenMM.

The speed of the method allows structural biologists to focus on the science of viral genome packaging. SU10 is a kuravirus that infects *Escherichia coli*; some kuraviruses have short replication cycles with many progeny, thus are candidates for phage therapy (11). For SU10, the DNA density in the cylindrical portion is clearly spiral. The end caps show concentric closed rings which appear to be rotational-averaging artifacts. We propose one structural hypothesis to explain the rings as well as a possible arrangement to connect to the spiral portion.

As a second example, we present our genome tracing for phage P68 (12). This is a quite different challenge, as the density is very noisy and full of artifacts. Initial attempts to fit the DNA manually failed to yield a consistent genome. However starting from a spiral ansatz (and dismissing as artifacts, those portions of the density that do not follow the spiral), we find packing consistent with expected interhelical and inter-shell spacings, as well as hexagonal packing, such as has been observed in other dsDNA viruses (2).

## Method

### General model building procedure

Our genome tracing and model building procedure is summarized in Figure 1.

It comprises the following steps:

- Examine the viral genome density. Identify regions that can be modeled as either spherical or cylindrical spirals. Some viruses can have both geometries in different regions.
- Choose an ansatz (spherical or cylindrical) for the first region. Choose approximate initial spiral parameters.
- Generate a coarse-grained genome model (one atom per base pair) and compute the fitting energy against the experimental density. Adjust the parameters manually if needed.
- Set up a loop in MMB to vary spiral parameters at fine granularity. Select parameters which minimize fitting energy. If varying important parameters does not change results, the density in the given region or winding shell may be too noisy.
- Once step 4 converges, convert the coarse-grained model to an all-atom model.
- If needed, anneal bonds using a Molecular Dynamics minimizer.
- Repeat steps 3–6 for each winding shell in the region.
- Go to step 2 and model the next region.
- If your model has two or more regions, make connections between regions manually using semiflexible fitting.

The coarse grained procedure (steps 2–4) is very fast. Many users who only wish to gain insight into genome organization will find coarse graining perfectly satisfactory.

The spherical spiral (in spherical coordinates) follows:


\begin{eqnarray*}\theta = \frac{p}{{r \cdot 2\pi }} \cdot \left( {\varphi - {\varphi }_o} \right)\end{eqnarray*}

where *θ* is the angle from the north pole, *φ* is measured about the north pole, *φ_0_* is the initial offset in the *φ* direction, and *p* is the pitch (distance between consecutive turns, measured along constant *φ*). Spherical spirals have the convenient property that the *p* is constant – as we would expect in the case of wound DNA. Similarly a cylindrical spiral (in cylindrical coordinates) obeys:


\begin{eqnarray*}z = \frac{p}{{2\pi }} \cdot \left( {\varphi - {\varphi }_0} \right)\end{eqnarray*}

MMB allows the user to manually set, in the spherical case, start and end angles *θ_start_* and *θ_end_*, in the cylindrical case the cylinder height *h*, and in both cases *r*, *p*, *φ_0_*, and the center *c* (expressed in *x, y, z*). MMB outputs one Mg^2+^ ion to represent each base-pair, in PDB (Protein Data Bank) or mmCIF format. This can be easily be superimposed on the density map using e.g. UCSF Chimera (13) for visual evaluation, and adjusted accordingly.

Manual adjustment should be followed by systematic scans over the parameters *r, c, p*, and *φ_0_*, optimizing fitting energy. For SU10’s outermost shell, here referred to as shell 1, no more than two rounds of optimization were needed. For shell 2 (the next shell inwards from shell 1) and inwards, only *r* and *φ_0_* change significantly, and the change in *r* is directly related to *d*, so only one round of optimization was needed.

### Fine graining

Fine-graining (step 5) is done by applying rigid-body translation-rotation transforms to idealized atomic models of DNA base pairs. The transforms build up an all-atom DNA model following the spiral, with the standard distance and relative rotation between consecutive base-pairs. This procedure is slower than the coarse-grained, but still takes only minutes. The rigid-body concatenation means that bonds connecting consecutive bases will be artificially compressed on the inside of the cylinder or sphere, and extended on the outside. Also the bending is assumed to be uniform rather than local, thus neglects variations in roll and tilt, and twist along helical turns (14). These effects are mild at larger winding radii, and more pronounced at small winding radii (close to the axis of the capsid). The bonds lengths can be annealed using a Molecular Dynamics (MD) minimizer.

The MD minimization (step 6) was done using our OpenMM python script, simulatePdb_OpenCL.py. As a prerequisite, the H5T and H3T atoms were added to the chain termini of each double-helical portion of the model. In the OpenMM script, the Adenine and Thymine base atoms (including the C1′ atom) were constrained to keep the base position unchanged and only the sugar-phosphate backbone atoms were allowed to move. The most probable DNA conformation, the canonical B-form, was induced by torsional restraints of δ, ϵ, ζ, β and γ backbone torsions, the χ glycosidic bond torsion, and the ν0 to ν4 torsions within the deoxy-ribose ring. The target values were set corresponding to the BB00 NtC conformer definition (15). The minimization converged within 200 steps using the OL15 DNA forcefield (16). The whole minimization of the SU10 (113720 residues) takes about one hour using the GPU-accelerated OpenCL platform of the OpenMM running on a single GeForce RTX 3090.

Manually connecting different spiral regions (step 9) can be the most labor-intensive part of the process. We will describe how this was done for SU10.

### Departures from ideal spherical and cylindrical geometry

Icosahedral capsids are faceted and depart from ideal spheres and cylinders. However for SU10 this error amounted (in the worst spots) to a fraction of a DNA helical diameter in the outermost DNA winding shell, which we will refer to as shell 1. The error became undetectable in shell 2 (the next shell inwards after 1), and shells 3, 4, etc. We did not correct this error in the cylindrical portion. In larger phages, this error could become significant. For that we have a feature which can vary any of the spiral parameters (in this case, mostly *r*) as a harmonic function of *φ*. According to the Fourier Theorem these can be added to compensate any perturbation. Alternatively the user can flexibilize a double-helical segment (or ‘window’, spanning several base pairs), and relaxing this segment into the local density, following (17). A loop can be written to scan the entire length of the viral genome in a moving-window method.

Although we did not allow the modeled bases leaving the ideal spheres or cylinders due to the low resolution of the density, already the minimization of the backbone atoms restrained towards the BB00 conformation revealed periodic perturbations in the ideal canonical BI DNA geometry. The conformation assignment of the resulting model using the DNATCO web service (18,19) (dnatco.datmos.org) shows regions where the DNA adopts nearly perfect BI (BB00) conformation followed by regions with less ideal BB00 conformation. These less ideal regions appear periodically every 10–11 nucleotides reminiscent of the periodic local appearance of the BII conformation explaining the bending and transcriptional silencing of DNA wrapping around a histone in the nucleosome particle (20).

### Connecting the cylindrical and end-cap portions of the SU10 DNA

Semiflexible fitting in MMB is a multiscale modeling process (21) which is highly manual and problem-dependent. The user should answer the following questions:

- What is your starting model?
- What part of your model needs to be flexible?
- What parts of your model are in danger of steric clashes? Select a clash prevention method (MD potential, or collision detecting spheres)
- What parts of your model need to be constrained? Should they be constrained to ground or to other parts of the molecule? Should the constraint involve only specific degrees of freedom, or all?
- Do you need to close gaps, for example turning two DNA helices into one?
- Do you need to enforce specific base pairs, stacking interactions, or backbone conformations?
- What parts of the model should be subject to a density-map fitting potential?

We modeled the connections between the end caps and the cylindrical portion manually. The connecting DNA was made flexible and pulled using springs to connect to corresponding DNA in the cylindrical portion (17). Clashes between adjacent DNA helices were prevented using collision-detecting spheres and ‘Physics where you want it’ (17). Elastic tethers were used to restrain the DNA to a sphere concentric with the end cap. Additional tethers were used to allow the circular-arc DNA to rotate only in-plane with its corresponding ring-shaped density.

### Tracing the P68 genome

P68 is a regular icosahedron, (12) not elongated like SU10. Thus a spherical spiral ansatz was used exclusively for genome tracing. Due to the high noise and many artifacts, it was particularly important not to use human judgement but rather strictly minimize fitting energy. At each local minimum of *r*, we optimized *φ_0_* before optimizing *r* again. For shells 2–5, where we found clearly defined optima for *r* and *φ*_0_, we generated all-atom DNA models (Figure 7).

## Results and discussion

### Cylindrical region of SU10

**Figure 2.**
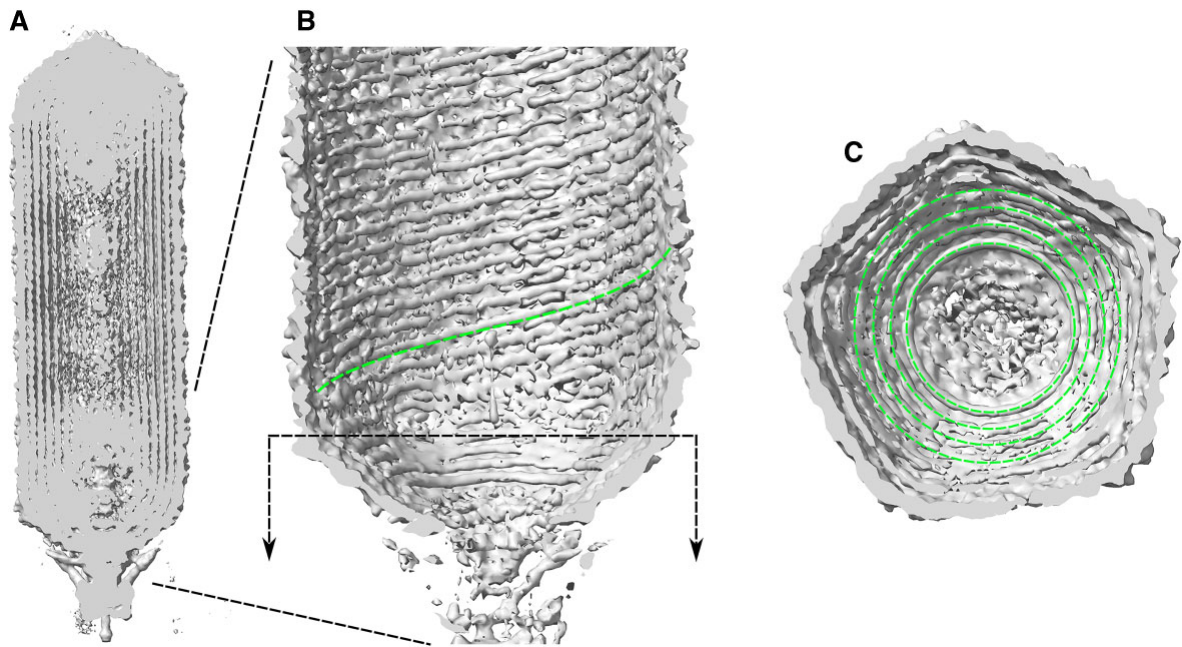
Views of the SU10 density showing traced genome. (**A**) Cutaway of entire virus shows DNA filling most space inside the capsid. Note concentric spherical domes of DNA density in the distal and proximal (near packaging motor) domes, and cylindrical packing in the long central portion. (**B**) Dashed green trace – example coarse-grained genome tracing. Note very high p, corresponding to *n*_pe_= 10 points of entry in cylindrical portion. Black dashed line with arrows: cutaway perspective of proximal end cap. (**C**) Proximal end cap. Green traces: *n*_pe_/2 – 1 = 4 circular hoops of traced genome density which relate capsid diameter to *n*_pe_, see text. Note the innermost ring (ring 4) is the most defined, and rings become less defined as diameter increases.

SU10 has an elongated icosahedral capsid (Figure 2). Most of the DNA is in the long central portion, which we call the cylindrical region. After optimization the cylindrical spiral of shell 1 had a slope of 12° and a *p* of 25.1 nm – quite large compared to the *d*= 2 nm diameter of DNA. This is because this region has n_pe_= 10 points of entry (analogous to a screw with 10 threads rather than the typical 1). Quickly arriving at such observations is an important contribution of our code.

### Intrashell versus intershell interhelical distances

**Figure 3.**
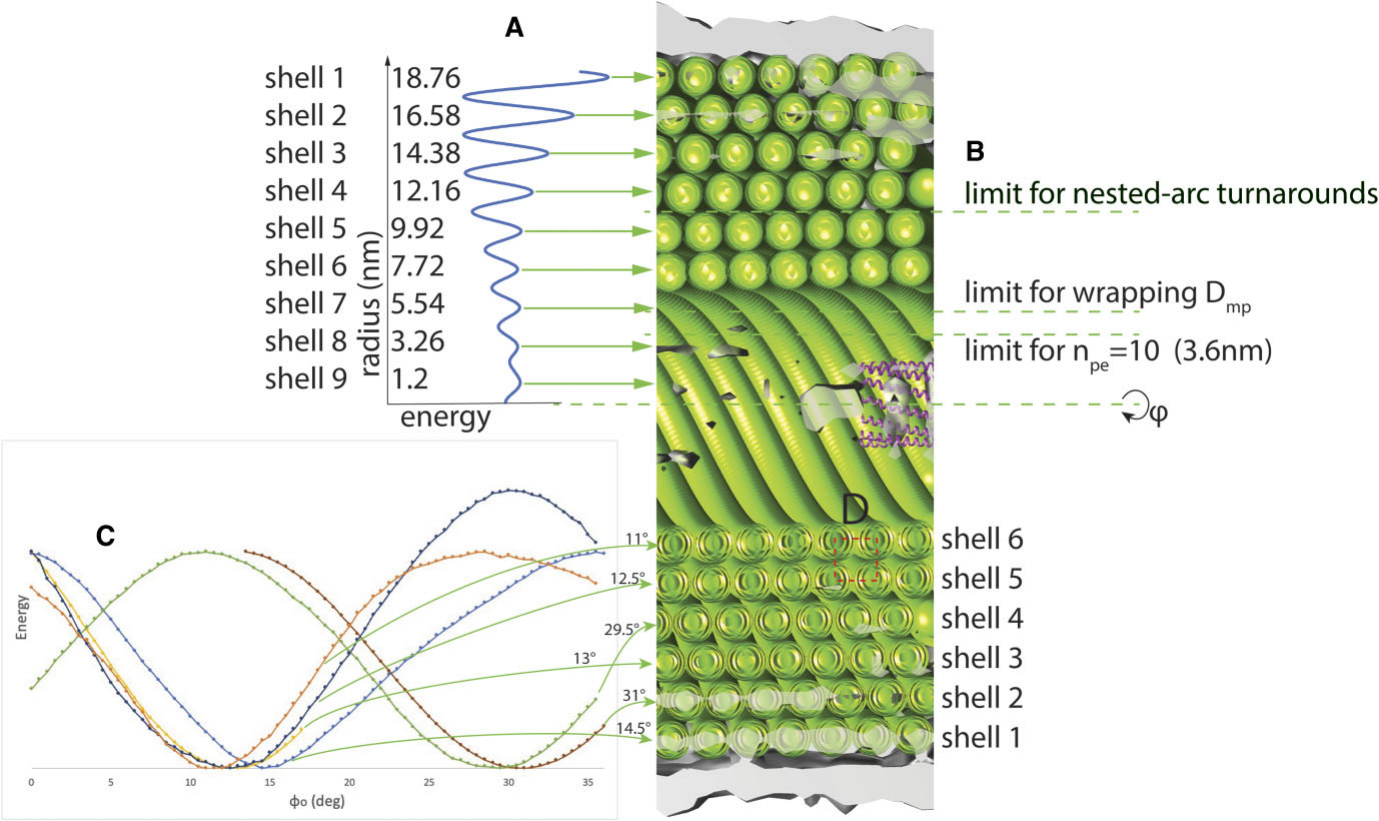
Optimizing *r* and φ_0_ for the cylindrical spiral DNA in SU10. (**A**) We fixed *n*_pe_= 10 and scanned *r*. The fitting energy shows DNA is organized in shells spaced 2.2 nm (average) apart, all the way down to a shell 9, even though visually, no DNA helices can be discriminated from shell 3 inwards. (**B**) At certain *r* values, winding behavior should change. Only shells 1–4 can have nested-arc turnarounds. At *r* < 3.6 nm, *n*_pe_= 10 becomes impossible, though optimizations for p, φ_0_ consistent with *n*_pe_= 10 fail to converge from shell 7 inwards. (**C**) For *n*_pe_= 10, traces are 36° apart. Therefore consecutive shells should have φ_0_ values 18° apart for hexagonal packing, and this approximately holds for shells 1–6. Puzzlingly, shells 5 and 6 have very similar φ_0_. (**D**) Cutaway view of part of the cylindrical portion of SU10, showing coarse-grain DNA models for shells 1–6.

The intrashell interhelical distance (perpendicular distance between DNA helices, measured between the helical axes) between adjacent strands comes to 2.46 nm for shell 1. The intershell spacing is Δ*r_inter_* = 2.18 nm between shells 1 and 2 (Figure 3). This gives an intershell interhelical distance of $\sqrt {{{( {\frac{{2.46\,{\rm nm}}}{2}} )}}^2 + 2.18\,{{\rm nm}}^2} = 2.50\,{\rm nm}$—slightly greater than the intrashell interhelical distance. However when we get to shells 4 and 5, we get Δ*r_inter_* = 2.24 nm and an intrashell interhelical distance (at shell 4) of 2.23 nm, giving again an intershell interhelical distance of 2.50 nm. Thus the difference between intrashell and intershell interhelical distances becomes greater as we move inwards (up to shell 5), because the intrashell interhelical distance steadily decreases while the intershell interhelical distance remains constant.

### Hexagonal packing in shells 1-5

We noted qualitatively that the DNA forms a hexagonal pattern, as indeed is expected for optimal packing. Since there are *n_pe_* = 10 points of entry, shell 2 should be rotated by $\frac{{360^\circ }}{{2 \cdot {n}_{pe}}} = 18^\circ$ with respect to shell 1, and this pattern should continue, which it does, at least within 2°—up to shell 5 (Figure 3). Interestingly shells 5 and 6 have very similar *φ*_0_, within 2°, and so are *not* packed efficiently against each other, despite having the same *n_pe_*.

### How many DNA turns are in the end-cap portions of SU10?

**Figure 4.**
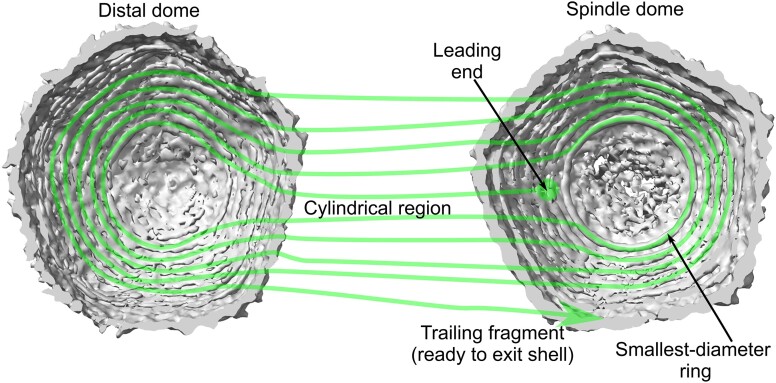
Proposed winding scheme of SU10, here shown for shell 1. We trace the DNA from the leading end (first terminus to be packaged into the capsid, green ball) towards the crossover fragment (last portion of DNA in shell 1). We describe the final packaged configuration, rather than any transient configurations. Lines are for reference only. In our model, the leading end attaches in the tail dome. From there, it forms one of the *n* = 10 points of entry and winds in a spiral fashion along the cylindrical portion. At the distal dome, it follows the smallest-diameter ring, then re-enters the cylindrical region, following parallel to and adjacent to the earlier spiral, to form the second point of entry. In the tail dome, it now follows the second-smallest-diameter ring, then reenters the cylindrical region. In the distal dome, it follows the second-smallest-diameter ring. Then it reenters the cylindrical region, adjacent to an earlier spiral. And so on. Once four rings in the tail dome, and five rings in the distal dome, have been filled, the DNA begins the 10th point of entry, and upon arriving at the tail dome, skips (green arrow) to begin filling shell

If *n*_pe_ (=10, here) is an even number, then the leading end should be in the same cap as the crossover where the DNA departs shell 1 and enters shell 2. If this happens at the tail dome, that means that there must be $\frac{{{n}_{pe}}}{2} = 5$ turns at the distal dome, and indeed approximately this many can be seen in the density, in the form of concentric rings. Following the same assumption, at the tail end there must be $\frac{{{n}_{pe} - 2}}{2} = 4$ turns, since the leading and crossover ends (see Figure 4) account for two of the *n*_pe_ points of entry. The volume occupied by the tail motor argues for the smaller number of turns to be in the tail dome. *n*_pe_ is a constant, at least for the first six shells in SU10 (Figure 3). We refer to this turnaround scheme as nested-arc turnarounds, and we will later argue it applies to shells 1 to 3 or 4. One evidence for this model is the circular densities of DNA—with higher noise for higher diameter consistent with a circular arc spanning an angle which decreases with diameter.

### Connecting SU10 cylindrical to dome DNA

**Figure 5.**
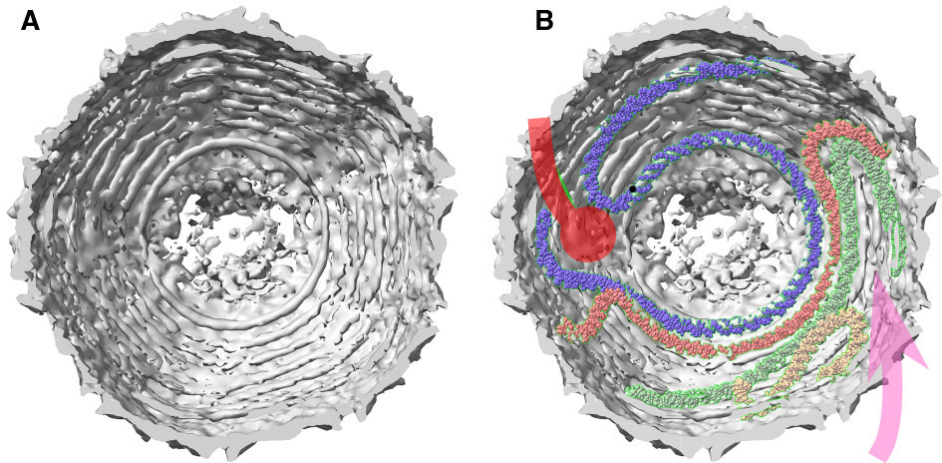
A possible connection scheme between the cylindrical and tail-dome portions. (**A**) One can arguably count four closed, concentric rings of DNA-like density in the tail dome, outermost shell. The innermost ring is very clear, while the remaining rings get lower contrast and/or more noise with increasing radius. The closed appearance of the rings is likely due to rotational averaging. (**B**) The increasing noise and 4 (=*n*/2 – 1) rings could be explained by the proposed connection scheme shown. The leading end (red) lodges somewhere in the tail dome. One stretch of DNA (purple) can then follow most of the innermost-ring density, except for a small portion as required to avoid said terminus, plus allow for smooth curvature as the DNA transitions to the cylindrical portion. The next turn (orange) cannot follow the second ring to an equivalent degree, because it needs to avoid the purple DNA. The following two turns (green and yellow) can follow their respective density rings to an even lesser degree.. The pink arrow represents the last portion of shell 1 DNA in the tail dome, transiting to shell 2. Each of the turns (purple, orange, green, yellow) has two ends, thus the four connect to eight stretches of DNA in the cylindrical portion of the virus. In total, these match the *n* = 10 stretches of DNA (points of entry) which form the cylindrical portion of the outermost shell.

Our suggestion for the connection between the cylindrical portion to the tail dome is illustrated in Figure 5. Note the concentric, full-circular density traces. As we elsewhere discuss, the DNA is unlikely to be circular, rather this is apparently a rotational-averaging artifact. The noise increases as radius increases – one explanation for this is that inner rings represent circular-arcs of DNA with greater arc-angle, while rings with larger radius span a smaller arc-angle. We propose that the innermost ring is connected via two closely-spaced DNA helices which connect to correspondingly closely-spaced strands in the cylindrical portion. These two strands are separated by only one stretch of DNA—the leading end (red). The second-smallest-diameter DNA arc (orange) cannot have as large an arc-angle, because its connecting DNA must leave space for the red and purple DNA. We semiflexibly connected the end-cap to the cylindrical DNA as explained in the Methods. The goal was that the connecting DNA should be restrained to the same spherical shell as the circular-arc DNA, should connect according to the above ansatz, and should not clash between helices.

### Do the points of entry and winding rules have to change for the inner shells?

Shells that conserve *n*_pe_ have a DNA slope (axial/tangential travel) that increases as diameter decreases. Since the maximum slope is infinity (DNA perfectly parallel to the virus axis) there is a minimum radius of (strand separation * *n*_pe_)/2π ≈ 3.7 nm for the innermost shell that would conserve *n*_pe_= 10. Density indicates shell 7 exists, and at ≈5 nm would be the smallest shell that could still preserve *n*_pe_= 10. However we could not find a combination of *n*_pe_, *p* and φ_0_ that convincingly explained the density for shell 7. The density indicates shells 8 and 9 exist, though these cannot preserve *n*_pe_= 10.

There is an additional reason why *n*_pe_= 10 cannot hold for all shells. Near-constant *p* is required for the DNA in a given shell to pack hexagonally with that of adjacent shells. However the slope *θ*_s_ of the DNA is a function of *p* and *r*: ${\theta }_s = {\tan }^{ - 1}\frac{p}{r}$, thus increases as *r* decreases. This means that the interhelical distance *d*_*ih*_ (measured perpendicular to the axes of adjacent DNA helices) is decreasing as *r* decreases: ${d}_{ih} = \frac{p}{{{n}_{pe}}}cos( {{\theta }_s} )$. *d_ih_* is 2.46 nm for shell 1, dropping to 2.23 nm for shell 6. In order for shell 7 to maintain *n_pe_*= 10, it would have to have *d_ih_*= 2.05 nm, a much tighter packing.

In summary, the turnarounds of shell 1 (Figures 4 and 5) would apply up through shell 3 and possibly 4, as earlier discussed, after which a new turnaround scheme would be needed. *n_pe_*= 10 appears to be respected up to shell 6, but cannot be after shell 7. Shells 7–9 appear to exist but we were unable to verify that they are packed in a spiral.

### Why is the base of the SU10 portal protein conical?

**Figure 6.**
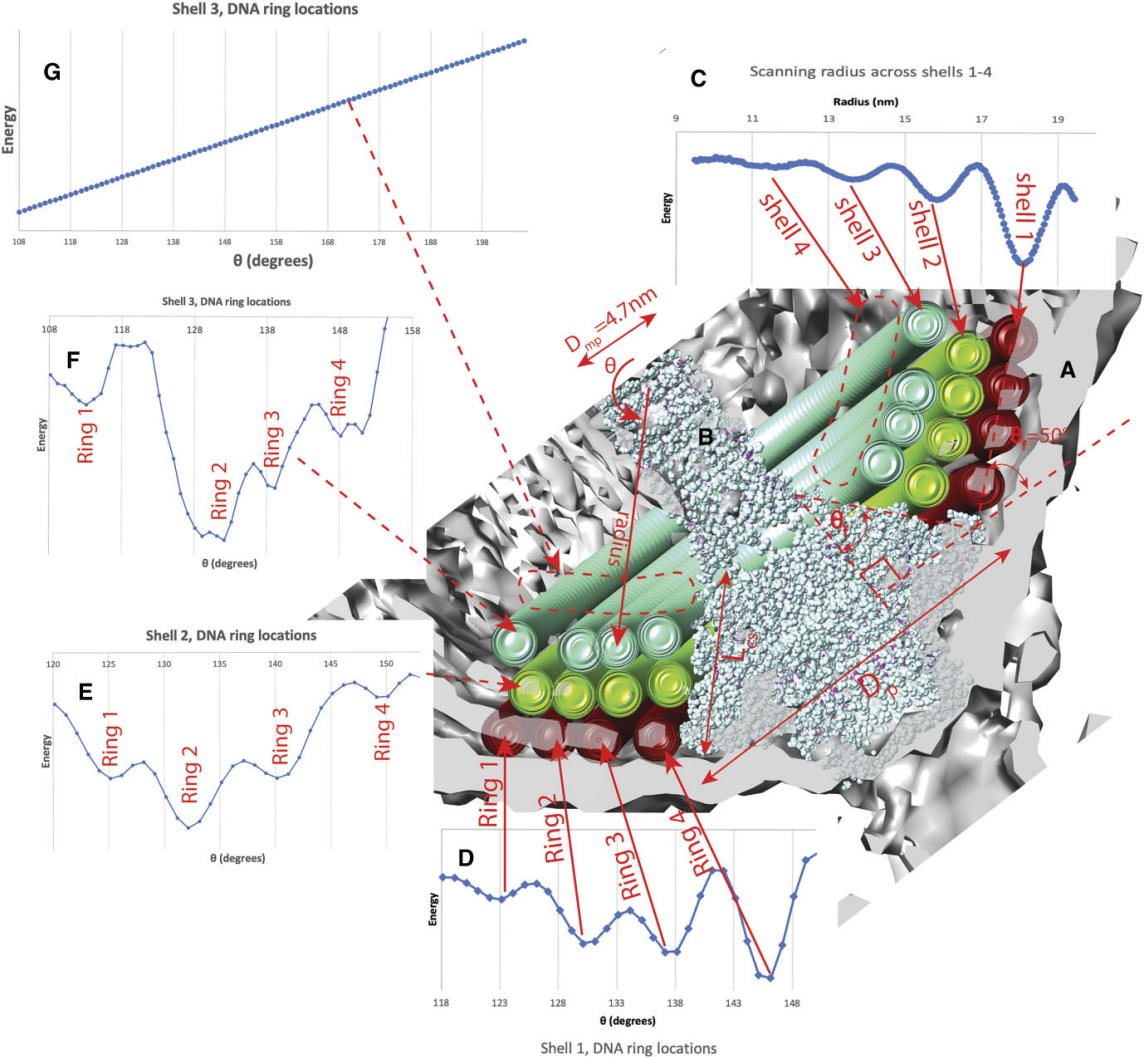
The capsid inner diameter is quantized in *n*_pe_ and *n*_s_, and the portal protein helps organize this. (**A**) Density map showing spindle dome of SU10. We used spherical coordinates for modeling, see markings to define radius and θ. (**B**) Portal protein (PDB ID: 7Z44). Note angle subtended by the conical portion is θ_f_= 51°, same as the angle formed by the shell 1 DNA. The length of the conical slope ${{\boldsymbol{L}}}_{{\boldsymbol{cs}}}$ is related to the number of shells obeying nested-arc turnaround rules, ${{\boldsymbol{n}}}_{\boldsymbol{s}}$, see formula in text. (**C**) Fitting energy (for rings 1–4) versus radius from center of modeled sphere. Note clear and sharp minimum for shell 1, becoming progressively less clear for shells 2 and 3, and quite unclear for shell 4. (**D**) Fitting energy for a single ring of DNA, in shell 1 while scanning over θ. Note Ring 4 is clearest. (**E**) Similarly, for shell2. (**F**) Similar, for shell 3. Note energy trace is noisier. G, For shell 4, it is impossible to discern any rings.

We argued that the $( {\frac{{{n}_{pe}}}{2}} ) - 1 = 4$ turns in the tail cap form concentric arcs about the portal protein (Figure 4). We propose that shells 1–3 and possibly ${n}_s = 4$ (*n_s_* is better defined below) follow the same rule, and that the taper in the portal protein is specifically designed to accommodate or even guide this. The idea is that the suspiciously smooth funnel shape of the portal protein presents a progressively decreasing diameter to shells 1 to 4, so that the (innermost) ring 1 can shrink in diameter as shells become progressively smaller. This is discussed in Figure 6 and below.

**Figure 7.**
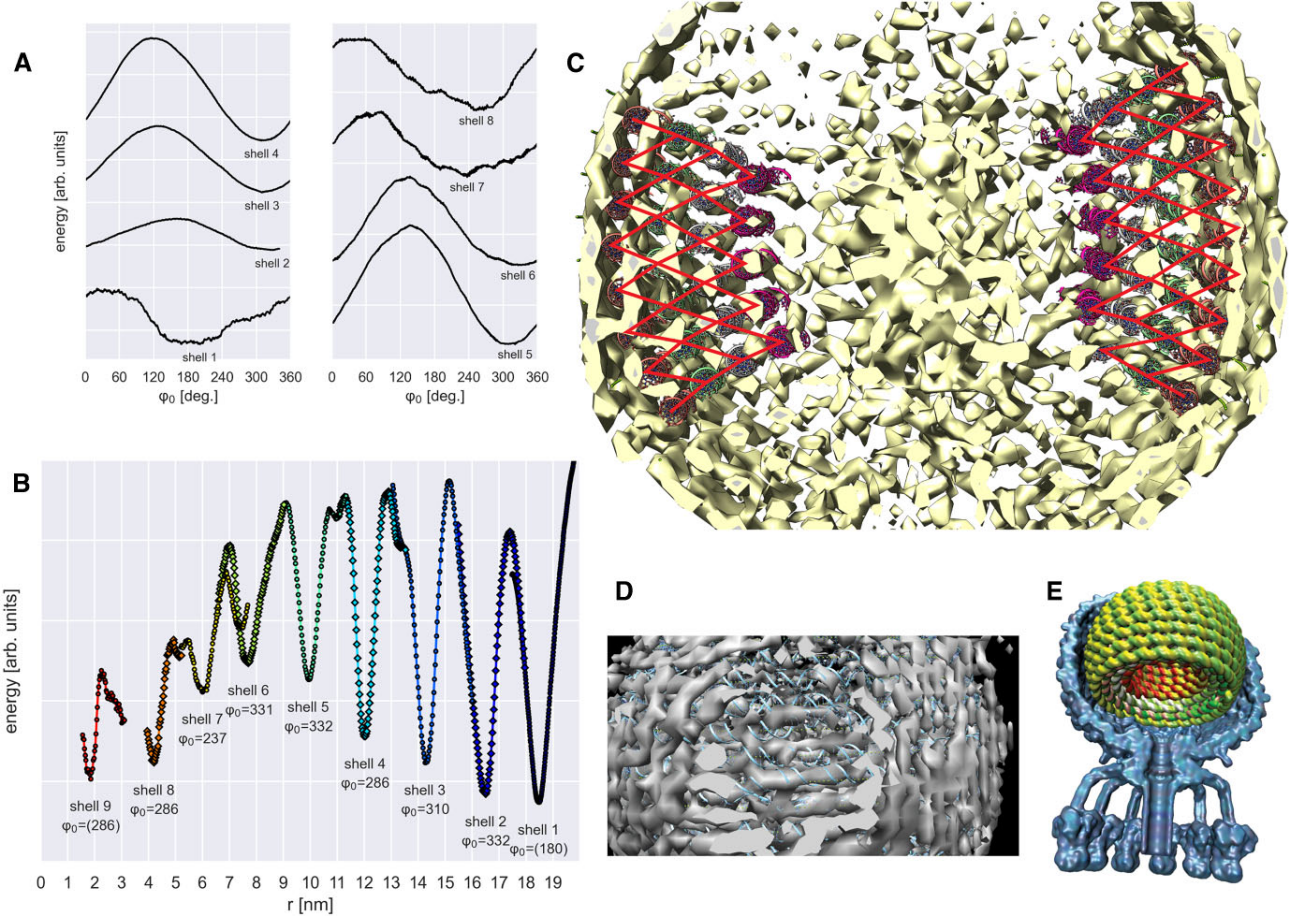
Genome tracing in the phage P68 density map, which has significant noise and artifacts. (**A**) Fitting energy versus φ_0_ (rotation about axis). Note clear minima in φ_0_ for shells 2–6. Noise makes φ_0_ optima unclear for shells 1, 7 and 8. (**B**) Energy versus *r*, using φ_0_ optimized for each shell. Energy minima are labelled with corresponding shell number, and optimal φ_0_ value—parentheses (shells 1 and 9) indicate highly imprecise values. Note radii even of shells 1 and 7–9 follow the separation of the better-resolved shells, on average 2.05 nm apart. (**C**) All-atom DNA shells 2–5, shown superimposed on density map. We did not attempt to fit near the poles due to high noise. Note that despite all shells being optimized independently, the packaging is approximately hexagonal, indicating that the spiral parameters are correct. (**D**) Close-up of our earlier, manual attempt at fitting DNA locally. No consistent single genome could be traced locally, leading to our conclusion that the true genome follows a spiral genometry, and the many departures from helicity are artifacts. (**E**) Cutaway of P68 capsid (PDB ID: 6Q3G) with our DNA model.

### Are *kuravirus* capsid inner diameters quantized under nested-arc turnaround rules?

The portal protein has several remarkable characteristics:

- Smooth protein surfaces are relatively rare in nature. And yet the portal protein presents a near-ideal funnel geometry, sloping smoothly from the major diameter *D_p_* (here 18 nm) to the minor diameter *D_mp_*.
- *D_mp_* = 4.7 nm is close to 4.1nm, the minimum radius of curvature of DNA as reported in (22), thus it could be a constant (or nearly so) among related viruses.
- The sloping portion has length *L_cs_* which well matches that required for spanning *n_s_* shells, if ${n}_s = 4$. After adjusting for boundary conditions this is: ${L}_{cs} = 8.5\,{\rm nm} = {n}_s \cdot 2.3\,{\rm nm} + {C}_{cs}$, where 2.3 nm is the intershell distance and ${C}_{cs} \approx - 2\,{\rm nm}$, adjusts for edge effects.
- Here the dome facets are equilateral triangles, and the slope of the funnel ${\theta }_f \approx 51^\circ$ is perpendicular to the dome facets such that it follows (or guides) the slope of the stacked inner rings of the *n_s_* shells.

The above imply that *D_p_* is quantized in *n_s_*, according to:


\begin{eqnarray*}{D}_p = 2 \cdot \left( {{{\mathrm{n}}}_s \cdot 2.3{\mathrm{nm}} + {C}_{cs}} \right) \cdot \sin \left( {{\theta }_f} \right) + {{\mathrm{D}}}_{mp}\end{eqnarray*}

Finally, the capsid inner diameter is quantized in *n_pe_, n_s_* as:


\begin{eqnarray*}&& 2 \cdot 2.48nm \cdot \left( {\frac{{{n}_{pe}}}{2} - 1} \right) \cdot \cos \left( {{\theta }_f} \right) \\ &&+ 2 \cdot \left( {{{\mathrm{n}}}_s \cdot 2.3{\mathrm{nm}} + {C}_{cs}} \right) \cdot \sin \left( {{\theta }_f} \right) + {{\mathrm{D}}}_{mp} + {{\mathrm{w}}}_{tr}\end{eqnarray*}

*w*_tr_ corrects approximately for the width of the transition between the spindle dome arcs and the cylindrical spiral. For SU10 *w_tr_*= 8.3 nm. If the proposed connection model is correct, this quantity could be driven by DNA bending strain.

The portal protein could be designed to accommodate a different *n_s_* without changing *n_pe_*, and *D_p_* would change in discrete steps. Thus *D_p_* is quantized in *n_s_*. Likewise the number of nested-arc turnarounds is quantized in *n_pe_*. Other members of the genus *kuravirus* may display different *n_s_* and *n_pe_* and in that case we would expect the inner capsid diameters to vary in discrete steps. There may also be a preference for specific values of *n_s_* and *n_pe_*, in which case many members would all have the same diameter.

### Is there experimental evidence that capsid diameters are quantized?

**Figure 8.**
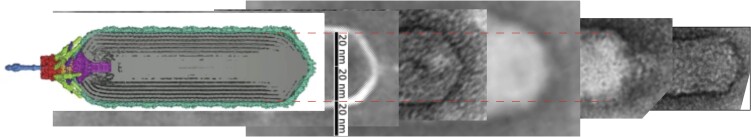
Size comparison of elongated-head phages. Phage images copied from the original publications, all with tails on the left, aligned with the boundary between tail dome and cylindrical portion.. The images were proportionately scaled together (using Adobe Illustrator) such that their100 nm scale bars matched in size for all. Left to right: SU10(11), Vp_R1 (Creative Commons license) (29), ΦGF1 (CC-BY-NC 4.0 International) (26), YF01 (CC BY) (27), pSal-SNUABM-01 (license #5631400182764) (24). phage 7–11 (license #5636721280470) (28). Red dashed lines are drawn from the inner diameter of SU10. The first four seem to have similar capsid inner diameters, with the possible exception of YF01 (which also seems to have lower resolution).) pSal-SNUABM-01 belongs to the family Podiviridae, but is the only one not of the genus Kuravirus (24).

The C3 morphotype (shown by kuraviruses) is rare (23); SU10 is the only example in the EMDB. There are, however, single-particle electron micrograph images of C3 morphotype viruses, which can be compared to SU10 (11), superimposed in Figure 8. Of these, kuraviruses SU10, ΦGF1, Vp_R1 and 7–11 have similar diameters, hinting at a preference for *n*_s_= 4 and *n*_pe_= 10. YF01’s diameter appears larger, however this may be down to this image's apparently lower resolution.

There is also virus pSal-SNUABM-01 which clearly has a smaller diameter than the others (24). While pSal-SNUABM-01 is in the family *Podiviridae*, it is reportedly not of the genus *Kuravirus*. The capsid cavity diameter appears to be about ≈75% that of SU10 (measured within Adobe Illustrator). If pSal-SNUABM-01 follows nested-arc turnaround rules, this could result from choosing *n_pe_*= 6 and *n_s_*= 3, *n_pe_*= 10 and *n_s_*= 1, or *n_pe_*= 4 and *n_s_*= 4.

The available electron micrographs are consistent with kuravirus capsid diameters being quantized in n_pe_ and n_s_, and hint at preferred values for these parameters.

### What does this say about slippage in the packaging motor?

The *n_pe_*= 10 is constant for cylindrical shells 1–6. Under our proposed model, the turnarounds in the end caps follow a similar scheme for shells 1–3 and perhaps 4. Thus even as shell diameter decreases, linking number remains constant, and therefore the linking number per base-pair, is changing across shells, which would favor a non-stoichiometric motor. However it is also not clear that each shell is filled completely before starting on the following shell, so unfortunately this question is not settled.

### What did we learn from P68?

The main take-away from P68 is that our method has significant ability to correct for artifacts and to detect the DNA geometry even when DNA cannot be distinguished visually. We found evidence of nine winding shells, each with a single point of entry, equally (2.05 nm on average) spaced, the smallest having *r =* 1.95nm (near the minimum possible). We believe the geometry is correct because it recapitulates approximately hexagonal packing (based on the clearest shells, #2–5) and equal shell spacing, despite each shell being optimized independently (Figure 7).

### Could other dsDNA viruses follow related principles?

**Figure 9.**
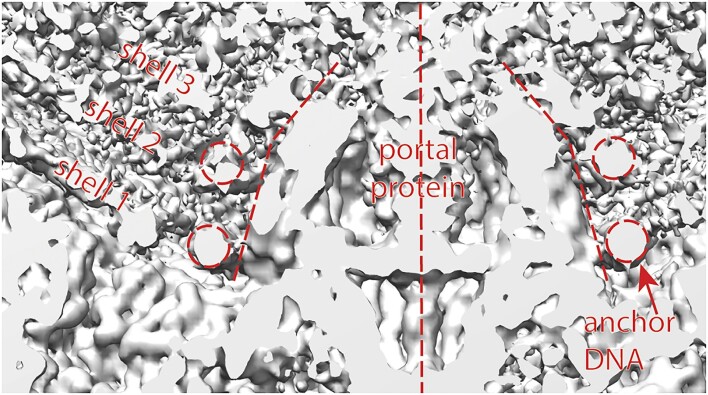
The portal protein of Herpes Simplex Virus (HSV)-1 may stabilize and guide the inner turns of DNA, analogous to SU10. There is a strikingly clear ring of density around the base of the portal protein, aligned with shell 1. Above that, there is a somewhat less-clear ring, aligned with shell 2. The portal protein appears to likewise have smooth walls, though in this case there appear to be two separate taper angles. Rendered using Chimera from EMD-9864(2).

While our work focuses on SU10 and the kuravirus family, the use of a precisely shaped portal protein to guide DNA wrapping around the spindle end is a simple idea.which could be implemented by other dsDNA viruses. Indeed Herpes Simplex Virus (HSV)-1 may be one example. The portal protein again has smooth sides, though this time seems to have two different taper angles (Figure 9). About the base of the protein appears (once again) a remarkably clear circular trace, so clear that Liu *et al.* dub it the ‘anchor DNA’(2) because it seems to be stabilized or ‘portal-anchored’, much like ring 4 of shell 1 of SU10 (Figure 6). One layer above that appears another circle, somewhat less clear, again much as occurs in SU10. The sloping walls of the portal protein this time are long enough in the radial direction to accommodate the first two winding layers, before changing taper.

We propose that a methodology similar to that employed for SU10 may uncover the packaging principles of HSV-1. In addition to the differences in portal proteins, HSV-1 is much larger than SU10, and also has a different capsid geometry. We therefore expect a different packaging scheme to emerge in future work.

## Conclusions

Building DNA models into electronic density maps of viruses is currently an extremely labor-intensive process, often requiring months of full-time work. However for many viruses including icosahedral ones, the DNA follows a spiral geometry. We have created a new MMB feature which automates many steps of model building for such cases. Coarse-grained DNA spirals can be generated and evaluated against experimental density maps, with each iteration taking seconds. The spiral DNA model for all distinguishable shells can be built in 1–2 days. It can even deduce the spiral parameters of winding shells that are far too noisy to be discriminated by eye, and we here show that those shells are consistent with those than can be visually verified. The coarse-grained model used for parameter optimization is easily upgraded into all-atom DNA.

Much more interestingly, the method speeds discovery. Our SU10 model suggests a sense in which capsid geometry is quantized with respect to the number of points of entry in the cylindrical portion, for viruses with SU10-like capsids and DNA winding schemes. This is because n_pe_ is directly related to the number of turns in the end caps. Further, the portal protein appears designed to set the number of shells (*n_s_*) that follow these turnaround rules. Together *n_pe_* and *n_s_* appear to set the capsid inner diameter. The fact that all three sufficiently-clear micrographs of other kuraviruses are very close in diameter provides experimental support for this.

The principles are simple and related ones may be applied in other viruses. Indeed HSV-1′s portal protein has smooth sides, albeith with two taper angles, and appears to stabilize DNA turnarounds, especially the smallest one of the outer shell (2). The similarities underscore one of the motivations of our work: the principles discovered in SU10 may apply to viruses of greater interest to human and animal health.

We point out that linking number is the same for all winding shells, but winding diameter thus base-pairs per shell get smaller as we progress inwards, and so finally linking number per base-pair is not constant across shells. It has has been suggested by (25) that each shell is filled prior to progressing to the next shell inwards; however this does not appear to be the case for e.g. P68 (12) and Harrison points out that it is incompatible with the presence of repulsive forces between adjacent helices (6). In the former case the packaging motor's twist per base-pair would have to vary during the packaging process – meaning slippage or other non-stoichiometric action.

Our method shows significant ability to correct for artifacts and discriminate structural details even in very noisy density maps. While we were unable to determine the turnarounds in P68, we were able to count the shells, show that *r* is regularly spaced, recapitulate hexagonal packing and determine *n_pe_* (for the clearest shells), and measure *φ_0_*, (albeit with widely varying accuracies).

We believe our method enables very strong time savings for structural virologists. The software is extremely economical, all work reported was performed on a single laptop core. Even more importantly, it enables virologists to make discoveries using very noisy density maps, well beyond the limit of what can be seen by eye or using atomistic fitting methods. We offer our code and assistance to the structural virology community. The method should also be compatible with future Machine Learning methods which would automate genome tracing. Such methods could be distributed via cloud services or a server.

## Data Availability

The spiral genome tracing feature is available in MMB 4.1.0, deposited as 10.5281/zenodo.10119019. Pulling the docker image samuelflores/mmb-ubuntu:4.1.0 provides the easiest access for most users. On Debian systems, MMB can be installed using the apt package manager. For advanced users, source (including the simulatePdb_OpenCL.py script) is available from github.com/samuelflores/MMB.git. MMB is under the open-source MIT license. The documentation in PDF can be separately obtained at http://pe1.scilifelab.se/MMB-annex/. The method can be applied by following the detailed instructions in the MMB Tutorial and Reference Guide from the above named version. MMB command scripts used to produce these results are available as supplementary information. The relevant SU10 density maps have EMDB accession numbers EMD-14492 for C1 and EMD-14488 for C5. The density map of P68 is deposited as 10.5281/zenodo.10036620. The all-atom 3D models of SU10 and P68 genomes in mmCIF format and the corresponding openMM minimization script are deposited as 10.5281/zenodo.10012813.
